# Detection of *parC* gene mutations associated with quinolone resistance in *Mycoplasma genitalium*: evaluation of a multiplex real-time PCR assay

**DOI:** 10.1099/jmm.0.001257

**Published:** 2021-02-19

**Authors:** Kaveesha Bodiyabadu, Jennifer Danielewski, Suzanne M. Garland, Dorothy A. Machalek, Catriona S. Bradshaw, Joshua Birnie, Samantha Ebeyan, Marie Lundgren, Gerald Murray

**Affiliations:** ^1^​ SpeeDx, Sydney, NSW, Australia; ^2^​ Centre for Women’s Infectious Diseases, The Royal Women’s Hospital, Parkville, VIC, Australia; ^3^​ Molecular Microbiology Research Group, Murdoch Children’s Research Institute, Parkville, VIC, Australia; ^4^​ The Department of Obstetrics and Gynaecology, University of Melbourne, Parkville, VIC, Australia; ^5^​ The Kirby Institute, University of New South Wales, Sydney, NSW, Australia; ^6^​ Melbourne Sexual Health Centre, Alfred Health, Carlton, VIC, Australia; ^7^​ Central Clinical School, Monash University, Melbourne, VIC, Australia

**Keywords:** *Mycoplasma genitalium*, antimicrobial resistance, fluoroquinolone, qPCR

## Abstract

**Introduction:**

Increasing levels of antibiotic resistance are complicating treatment for the sexually transmitted pathogen *Mycoplasma genitalium *. Resistance to fluoroquinolones is associated with mutations in the *parC* gene. Although the precise mutations conferring resistance are not fully understood, the single nucleotide polymorphism (SNP) G248T/S83I is most implicated.

**Aim:**

To evaluate the performance of the MG+*parC*(beta2) assay (SpeeDx, Australia), which detects single nucleotide polymorphisms (SNPs) in the *parC* gene at amino acid position S83 (A247C/S83R, G248T/S83I, G248A/S83N) and D87 (G259A/D87N, G259T/D87Y, G259C/D87H).

**Methods:**

Clinical samples were analysed by MG+*parC*(beta2) assay and results compared to Sanger sequencing. Sensitivity, specificity, and predictive value for treatment failure were calculated.

**Results:**

From analysis of 205 samples, the MG+*parC*(beta2) assay performed with a high sensitivity 98.2% (95% CI:90.3–100) and specificity 99.3% (95% CI:96.3–100) for *parC* SNP detection with a kappa of 0.97 (95% CI:0.94–1.00). The predictive value of G248T/S83I detection (the most common SNP, prevalence of 13% in the study population) was analysed with respect to treatment failure (patients received sequential doxycycline-moxifloxacin). The positive-predictive-value for moxifloxacin failure after detection of S83I was only 44% (95% CI:24.4–65.1), while negative-predictive-value was high at 96.9% (95% CI:92.7–99.0), suggesting that other SNPs are contributing to resistance.

**Conclusion:**

MG+*parC*(beta2) performed with high concordance compared to Sanger sequencing. Such qPCR assays can assist in understanding causes of treatment failure, inform the development of diagnostic assays, and can be applied to surveillance of mutations in populations. Due to an incomplete understanding of the basis for fluoroquinolone resistance, such tests do not appear to be ready for clinical application.

## Introduction

*Mycoplasma genitalium* is a sexually transmitted pathogen associated with a number of syndromes in males and females [1, 2]. Treatment of infection is complicated by increasing levels of resistance to the recommended first line treatment, the macrolide azithromycin, and second line treatment, the fluoroquinolone moxifloxacin [3].

Single nucleotide polymorphisms (SNPs) that confer resistance to azithromycin are well characterised, and tests reporting detection of *M. genitalium* and markers for resistance [4] have led to improved therapeutic outcomes through resistance-guided therapy [5, 6]. In contrast, the contribution of specific SNPs to fluoroquinolone-resistance remains unclear; associations have been drawn with SNPs in the *parC* gene resulting in changes at amino acid position S83 (A247C/S83R, G248T/S83I) [7–9] and D87 (G259A/D87N, G259T/D87Y) [10]. Of these, G248T/S83I is the most strongly implicated in resistance, however, approximately 45% of patients with infections exhibiting this SNP experience cure with fluoroquinolone treatment [7, 9]. Additional SNPs, not clearly linked with treatment failure, have also been described. These include G259C/D87H [11] and G248A/S83N (more common in Europe) [12–14].

Research tools that detect SNPs can help to develop an understanding of causes of treatment failure and inform the development of diagnostic assays. Quantitative PCR-based assays are generally quicker, more convenient, and cheaper than Sanger sequencing. We previously evaluated the test MG+*parC*(beta) (SpeeDx, Sydney, Australia) that detects *M. genitalium* plus five *parC* SNPs (excluding G248A/S83N) [15]. In this study we evaluate the performance of the revised assay, MG+*parC*(beta2), that detects the six above mentioned SNPs in *parC* (Table 1).

**Table 1.** Summary of the MG+*parC*(beta2) assay

Reagent mix	Target*	Amino acid change	Fluorophore†	Detection filters (nm)
Mix 1	G248T	S83I	Texas Red	618–660
	A247C	S83R	Texas Red	618–660
	G248A	S83N	JOE	533–580
	MgPa	na	FAM	465–510
	Internal control	na	Cy5	533–610
Mix 2	G259T	D87Y	Texas Red	618–660
	G259A	D87N	Texas Red	618–660
	G259C	D87H	Texas Red	618–660
	MgPa	na	FAM	465–510

*The assay target was either a single nucleotide polymorphism affecting the QRDR of *parC*, internal control, or the MgPa gene (for *M. genitalium* detection). Reference genome for indicated changes, *M. genitalium* G37, GenBank accession number NC_000908. Numbering refers to *M. genitalium* gene numbering.

†Texas Red, sulforhodamine 101 acid chloride; JOE, 6-carboxy-4′,5′-dichloro-2′,7′-dimethoxyfluorescein; FAM, 6-carboxyfluorescein dye.

## Methods

### Samples used for this study

*M. genitalium *-positive samples (*n*=242), were previously collected from patients undergoing sequential doxycycline-moxifloxacin treatment for macrolide-resistant *M. genitalium* [6]. The samples included 223 pre-treatment samples (a diagnostic sample taken prior to any antibiotic therapy) and 19 test of cure samples. Sample types included 166 urine, and 45 anal, 14 cervical, 14 vaginal/high vaginal, and two urethral swabs, plus one swab of unknown site.

### Analysis methodology

**Fig. 1.**
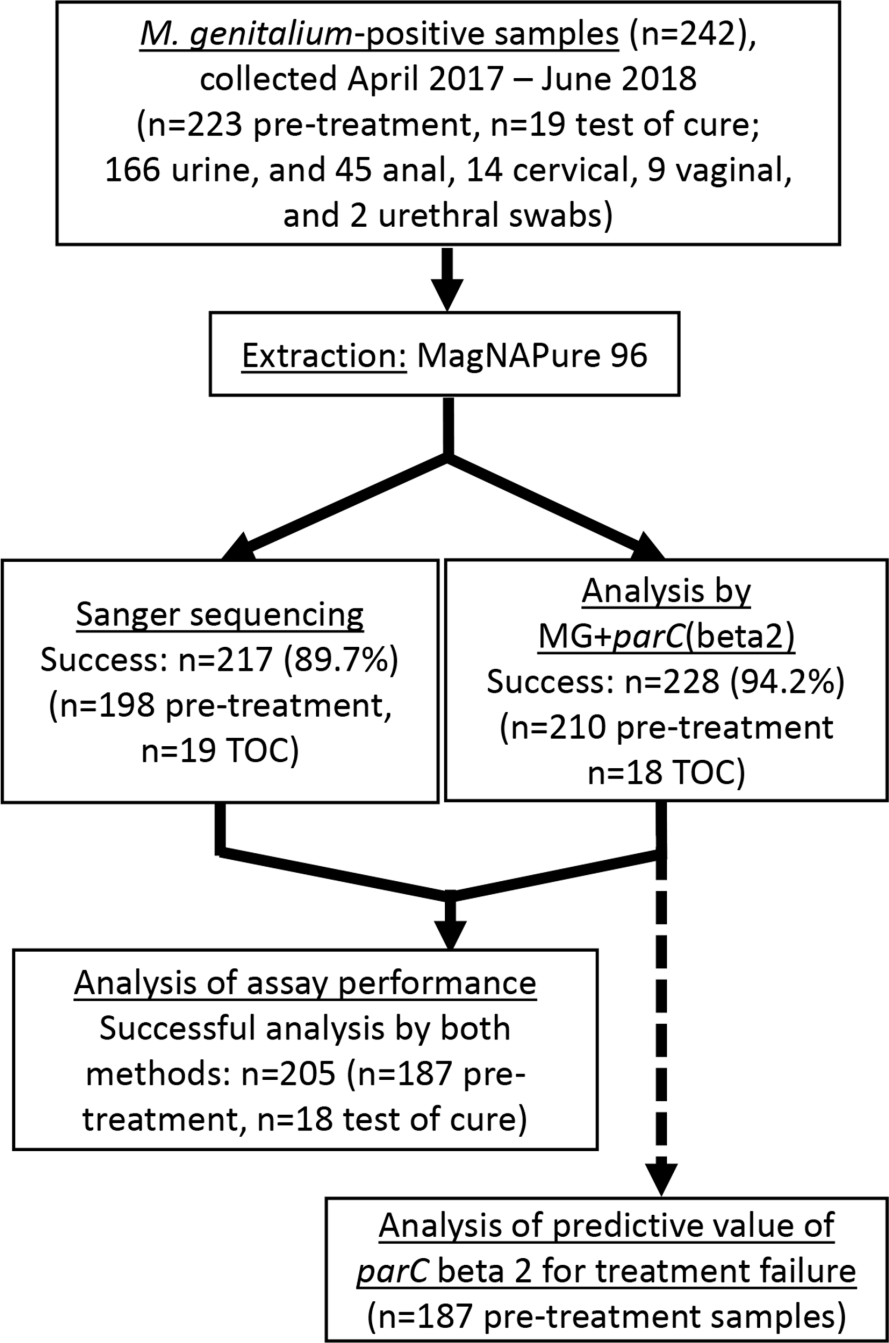
Overview of the *M. genitalium *-positive samples used for this study, and methods of analysis. TOC, test of cure.

Samples were extracted on the MagNA Pure 96 instrument using the DNA and Viral NA Small Volume Kit (Roche) with 100 µl elution volume [9]. To perform the MG+*parC*(beta) assay (manufactured by SpeeDx Pty Ltd.), master mix (15 µl) was mixed with sample extract (5 µl) and analysed on the LightCycler 480 II (Roche Diagnostics) with the following cycling conditions: one cycle of polymerase activation (95 °C, 2 min, ramp 4.4 °C s^–1^), 10 cycles of touchdown cycling (0.5 °C per cycle, 61–56.5 °C, 30s per cycle, ramp 2.2 °C s^–1^) followed by 40 cycles of quantification cycling/data acquisition (95 °C, 5s, ramp 4.4 °C s^–1^; 52 °C 40s 2.2 °C s^–1^), with a final step of cooling (40 °C, 30s, 2.2 °C s^–1^). Data acquisition was performed using filter combinations indicated (Table 1). Results for the MG+*parC*(beta2) assay were compared to the gold standard of Sanger sequencing [9] (Fig. 1).

## Results and discussion

### Performance of the MG+*parC*(beta2) assay compared to Sanger sequencing

A total of 205 out of 242 (84.7%) *M *. *genitalium* samples were successfully analysed by both methods (187 of these were pre-treatment samples). Sanger sequencing identified mutations targeted by the MG+*parC*(beta2) assay in 55 samples (*n*=40 G248T/S83I, *n*=13 G259A/D87N, *n*=2 G248A/S83N). The MG+*parC*(beta2) assay performed with a high sensitivity 98.2% (95% CI:90.3–100) and specificity 99.3% (95% CI:96.3–100) for *parC* SNP detection with a kappa of 0.97 (95% CI: 0.94–1.00) (Table 2).

**Table 2.** Comparison of single nucleotide polymorphism (SNP) detection for the MG+*parC*(beta2) assay compared to Sanger sequencing*

		Sanger sequencing		
		Mutant	Wild-type	Total
MG+*parC*(beta2)	Mutant	54†	1‡	55
	Wild-type	1§	149	150
	Total	55	150	

*Both baseline and test of cure samples are included.

†The 40 G248T/S83I SNPs (all detected by assay), 13 G259A/D87N mutations (12/13 detected by assay), 2 G248A/S83N mutations (all detected by assay). SNPs in the *parC* quinolone-resistance-determinant region not targeted by this assay include A260G/D87G, A247T/S83C.

‡Determined to be WT by sequencing, but G259A/D87N by MG+*parC*(beta2).

§Determined to be G259A/D87N by sequencing.

### Analysis of results in the context of treatment outcomes

The treatment outcomes for the patients in this study group are known, with 92% of patients cured by sequential doxycycline-moxifloxacin treatment, and 8% failing treatment [6]. This allowed calculation of the positive predictive value of the assay for treatment failure. The most common SNP in the study population was G248T/S83I, present at 13% of pre-treatment samples. The positive-predictive-value of the detection of S83I for treatment failure was determined by focusing on the assay channel reporting S83I/S83R (Table 1; note that there were no S83R SNPs in the study). From 187 pre-treatment samples the positive-predictive-value of S83I detection for moxifloxacin failure (which followed a week of doxycycline) was only 44 % (95% CI:24.4–65.1), while negative-predictive-value was high at 96.9% (95% CI:92.7–99.0). Other SNPs were rare in the study population. G259A/D87N was the next most prevalent, present in 6% of successfully analysed baseline samples. However, this mutation was equally represented in cures and failures.

The *parC* SNP G248T/S83I is the most strongly implicated mutation in *M. genitalium* fluoroquinolone resistance [7–9]. The results of this study indicate that detection of S83I has a low predictive value for moxifloxacin failure (particularly when used following doxycycline) and suggests that fluoroquinolone resistance may have a more complex basis than macrolide resistance, as outlined in the Introduction. Notably, in this study the pre-treatment of patients with doxycycline prior to moxifloxacin may have impacted cure, so the findings here do not necessarily translate to the situation where preceding doxycycline is not used. However, it is also important to note that quinolone resistance assays are likely to be used in the context of sequential doxycycline-moxifloxacin treatment as doxycycline is recommended within sequential treatment for *M. genitalium* in Australia and the UK [16, 17], and doxycycline is recommended for initial treatment of NGU (Europe, UK, Australia) [16–18], and as one of two recommended regimens for NGU in the USA [19].

As mentioned above, resistance to fluoroquinolones appears to be more complex than the situation for macrolides. There are other SNPs that are not captured by current *parC* assays that may contribute to resistance. These include SNPs affecting amino acids M95 and D99 in *gyrA*, which may have an additive effect when combined with *parC* SNPs [9]. Undefined variations elsewhere in the genome may also contribute. The results of this study demonstrate current limitations in using *parC*-based assays to triage patients away from moxifloxacin treatment while we still don’t have a clear understanding of mechanisms of resistance [20]. This is particularly important in an environment where there are limited alternative treatments for *M. genitalium *.

This MG+*parC*(beta2) assay was used in an earlier study using a smaller study group [21]. Sanger sequencing wasn’t used to confirm mutations, which were few in number (*n*=7), and treatment data were only available for three patients, so it is difficult to draw comparisons with the current study.

### Study limitations

Lower load samples are less likely to yield a successful sequencing result, so it is possible that they were not included in the analysis. Additionally, results for Australian samples may not be generalizable to other locations.

## Conclusion

MG+*parC*(beta2) showed very high specificity and sensitivity for detection of SNPs potentially associated with fluoroquinolone resistance. Such quantitative PCR-based assays are generally quicker and cheaper than Sanger sequencing, making this assay a useful tool for detecting *parC* mutations to investigate treatment failure and for the surveillance of mutations in populations.
